# TXH11106: A Third-Generation MreB Inhibitor with Enhanced Activity against a Broad Range of Gram-Negative Bacterial Pathogens

**DOI:** 10.3390/antibiotics11050693

**Published:** 2022-05-20

**Authors:** Eric J. Bryan, Hye Yeon Sagong, Ajit K. Parhi, Mark C. Grier, Jacques Y. Roberge, Edmond J. LaVoie, Daniel S. Pilch

**Affiliations:** 1 Department of Pharmacology, Rutgers Robert Wood Johnson Medical School, Piscataway, NJ 08854, USA; ejb236@dls.rutgers.edu; 2 TAXIS Pharmaceuticals, Inc., Monmouth Junction, NJ 08852, USA; hyeyeon.sagong@gmail.com (H.Y.S.); ajit.parhi@gmail.com (A.K.P.); 3 Department of Molecular Design and Synthesis, Rutgers University Biomedical Research Innovation Cores, Piscataway, NJ 08854, USA; mg1685@research.rutgers.edu (M.C.G.); jr1257@research.rutgers.edu (J.Y.R.); 4 Department of Medicinal Chemistry, Ernest Mario School of Pharmacy, Rutgers—The State University of New Jersey, Piscataway, NJ 08854, USA; elavoie@pharmacy.rutgers.edu

**Keywords:** bactericidal efficacy, *Escherichia coli*, *Klebsiella pneumoniae*, *Acinetobacter baumannii*, *Pseudomonas aeruginosa*, noncompetitive inhibition of MreB ATPase activity

## Abstract

The emergence of multi-drug-resistant Gram-negative pathogens highlights an urgent clinical need to explore and develop new antibiotics with novel antibacterial targets. MreB is a promising antibacterial target that functions as an essential elongasome protein in most Gram-negative bacterial rods. Here, we describe a third-generation MreB inhibitor (TXH11106) with enhanced bactericidal activity versus the Gram-negative pathogens *Escherichia coli*, *Klebsiella pneumoniae*, *Acinetobacter baumannii*, and *Pseudomonas aeruginosa* compared to the first- and second-generation compounds A22 and CBR-4830, respectively. Large inocula of these four pathogens are associated with a low frequency of resistance (FOR) to TXH11106. The enhanced bactericidal activity of TXH11106 relative to A22 and CBR-4830 correlates with a correspondingly enhanced capacity to inhibit *E. coli* MreB ATPase activity via a noncompetitive mechanism. Morphological changes induced by TXH11106 in *E. coli*, *K. pneumoniae*, *A. baumannii*, and *P. aeruginosa* provide further evidence supporting MreB as the bactericidal target of the compound. Taken together, our results highlight the potential of TXH11106 as an MreB inhibitor with activity against a broad spectrum of Gram-negative bacterial pathogens of acute clinical importance.

## 1. Introduction

Ever since their discovery in the early 20th century, antibiotics have been the principal tools for treating bacterial infections. However, over the past several decades, antibiotic resistance has risen alarmingly [1,2]. The World Health Organization (WHO) has identified numerous factors underlying the rise in resistance, including (i) overprescribing of antibiotics, (ii) failure of patients to complete their prescribed course of antibiotic treatment, (iii) overuse of antibiotics in livestock and fish farming, (iv) inadequate infection control measures in healthcare facilities, (v) poor hygiene and sanitation conditions, and (vi) an insufficient pipeline of new antibiotics targeted for clinical development [3]. Multi-drug-resistant (MDR) bacteria are becoming increasingly difficult to treat and greatly increase the chance of mortality, posing a significant threat to global public health [4,5]. In a recent report, the Centers for Disease Control and Prevention (CDC) have indicated that in the United States alone, over 2.8 million antibiotic-resistant infections occur each year, resulting in more than 35,000 deaths [6]. The majority of resistant bacterial pathogens designated by the CDC as being of particularly urgent concern are Gram-negative species [6]. Gram-negative pathogens are especially difficult to treat due to multiple resistance mechanisms that include an outer membrane that impedes drug influx, the expression of pumps that promote drug efflux, and the production of antibiotic-inactivating enzymes [7,8]. Among the MDR Gram-negative pathogens for which new treatment are urgently needed are *Acinetobacter baumannii*, *Pseudomonas aeruginosa*, and various Enterobacteriaceae (including *Escherichia coli* and *Klebsiella pneumoniae*) [5,6].

Addressing the threat posed by MDR bacterial pathogens requires the development of new antibiotics. The majority of antibiotics in current clinical use target bacterial processes underlying cell wall, protein, or DNA synthesis [9]. This limited scope of drug targets highlights the need for identifying new antibacterial targets and developing drugs that exploit them. Several promising antibacterial targets have been recently identified, including the outer membrane proteins LptD and BamA, the divisome protein FtsZ, and the elongasome protein MreB [10,11,12,13].

MreB is a highly conserved essential protein directly involved in cell wall formation and maintenance of most rod-shaped bacteria [14,15]. MreB is similar in both structure and function to the eukaryotic cytoskeleton protein actin, self-polymerizing into filaments in an ATP-dependent manner [15,16]. These filaments localize to the inner cell membrane where they participate in lateral cell wall synthesis of rod-shaped bacteria. MreB carries out this function through interactions with penicillin-binding protein 2 (PBP2) and other protein factors that comprise the elongasome [17]. Loss of functional MreB causes rod-shaped bacteria to grow as spheres, consistent with its function being required for cell elongation [18,19]. Elongasome proteins, including MreB and PBP2, are also directly involved in early bacterial cell division [20]. They transiently colocalize to midcell with divisome protein FtsZ, where they are postulated to facilitate synthesis of the septal peptidoglycan layer [20]. As many MDR bacteria of concern are Gram-negative bacilli, MreB is an especially promising antibacterial target [6].

**Figure 1:**
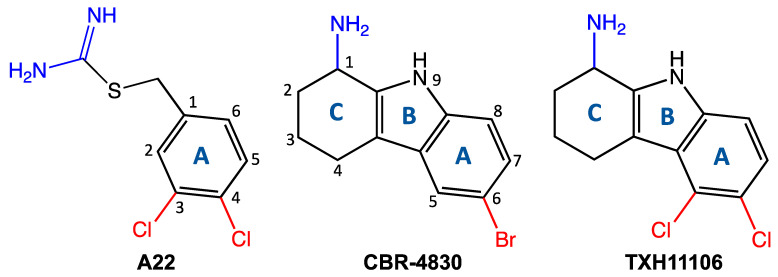
Chemical structures A22, CBR-4830, and TXH11106, with the lettering and atomic numbering of the rings being as indicated. The basic functionalities of each compound are highlighted in blue and the halogen substituents on ring A of each compound are highlighted in red.

Iwai et al. identified the benzylisothiourea derivative A22 (Figure 1) as an inhibitor of cell elongation that inhibits *E. coli* growth [21]. A22 has since been shown to bind and inhibit the polymerization of MreB, resulting in spherical-shaped Gram-negative bacilli characteristic of the phenotype associated with loss of MreB function [21,22,23]. Löwe and coworkers recently solved the crystal structure of A22 in complex with *Caulobacter crescentus* MreB (CcMreB) [23]. This structure revealed A22 to bind a site adjacent to the nucleotide binding pocket, with the latter being occupied by ADP [23]. While A22 has been established as an MreB inhibitor, it is generally associated with moderate antibacterial activity [22]. Robertson et al. identified CBR-4830 (Figure 1) as a second-generation MreB inhibitor that, like A22, induces a spheroid cell phenotype and inhibits MreB polymerization [24]. CBR-4830 exhibited enhanced activity versus *P. aeruginosa* relative to A22 [22,24].

In this study, we describe TXH11106 as a third-generation MreB inhibitor that incorporates the dichloro-substituted ring functionality of A22 (ring A) and the cyclohexylamine substituent (ring C) of CBR-4830 (see structures in Figure 1). Compared with A22 and CBR-4830, TXH11106 exhibits enhanced bactericidal activity against a broad range of clinically important Gram-negative pathogens, including *E. coli*, *K. pneumoniae*, *A. baumannii*, and *P. aeruginosa*. The enhanced antibacterial activity of TXH11106 correlates with a correspondingly enhanced inhibition of *E. coli* MreB ATPase activity, with this inhibition being non-competitive in nature. Significantly, TXH11106 is also associated with a very low frequency of resistance (FOR). In the aggregate, our results highlight TXH11106 as a lead MreB inhibitor worthy of further development.

## 2. Materials and Methods

### 2.1. Bacterial Strains and Other Reagents

*E. coli* ATCC 25922, *K. pneumoniae* ATCC 13883, *A. baumannii* ATCC 19606, and *P. aeruginosa* ATCC 27853 were obtained from the American Type Culture Collection (ATCC). A22 was obtained from Cayman Chemical Company, and CBR-4830 was synthesized as described previously [24]. Luria–Bertani (LB) media, cation-adjusted Mueller Hinton (CAMH) media, and tryptic soy agar (TSA) were obtained from Becton-Dickson. Ampicillin sodium salt was obtained from VWR, phosphate-buffered saline (PBS) was obtained from Lonza, and adenosine 5’-triphosphate (ATP) disodium salt hydrate was obtained from Sigma-Aldrich (St. Louis, MO, USA).

### 2.2. Synthesis of TXH11106 (***4***)

**Scheme 1:**
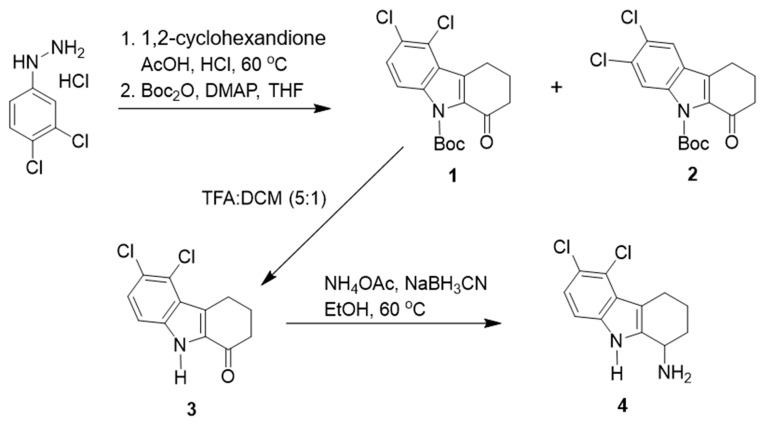
General scheme for the synthesis of TXH11106 (**4**).

As depicted in Scheme 1, 5,6-dichloro-2,3,4,9-tetrahydro-1*H*-carbazol-1-one (**3**) (100 mg, 0.39 mmol), ammonium acetate (301 mg, 3.90 mmol), and sodium cyanoborohydride (123 mg, 1.90 mmol) were dissolved in ethanol (10 mL). The reaction mixture was stirred overnight at 60 °C. After removal of solvent, the reaction mixture was then diluted with ethyl acetate. The organic layer was washed with 10% sodium hydroxide and brine, and then dried over sodium sulfate. The organic layer was then concentrated under reduced pressure. The residue was purified on an ISCO chromatograph (0–10% methanol/dichloromethane + 0.1% NH_4_OH) to give the product 5,6-dichloro-2,3,4,9-tetrahydro-1*H*-carbazol-1-amine (TXH11106) (**4**) as a white solid (45 mg, 45%). ^1^H NMR (300 MHz) (CD_3_OD) δ 7.18 (d, *J* = 8 Hz, 1H), 7.09 (d, *J* = 9 Hz, 1H), 4.08–4.03 (m, 1H), 3.04–2.98 (m, 2H), 2.15–1.99 (m, 2H), 1.83–1.75 (m, 1H), 1.72–1.65 (m, 1H); LC/MS RT = 2.72 (M-H^-^: 253/255).

The requisite intermediates were prepared as follows:

(i) 5,6-Dichloro-2,3,4,9-tetrahydro-1*H*-carbazol-1-one (**3**):

To a solution of *tert*-butyl 5,6-dichloro-1-oxo-1,2,3,4-tetrahydro-9*H*-carbazole-9-carboxylate (**1**) (450 mg, 1.27 mmol) in dichloromethane (20 mL), trifluoroacetic acid (4 mL) was added. The reaction mixture was stirred for an hour at room temperature. After removal of solvent, the mixture was diluted with ethyl acetate and was washed with 10% sodium hydroxide and brine. The organic layer was then dried over sodium sulfate and concentrated under reduced pressure to give the product (**3**) as a white solid (270 mg, 84%). ^1^H NMR (300 MHz) (CDCl_3_) δ 7.90 (d, *J* = 9 Hz, 1H), 7.47 (d, *J* = 10 Hz, 1H), 3.33 (t, *J* = 6 Hz, 2H), 2.64 (t, *J* = 6 Hz, 2H), 2.24 (t, *J* =6 Hz, 2H).

(ii) *tert*-Butyl 5,6-dichloro-1-oxo-1,2,3,4-tetrahydro-9*H*-carbazole-9-carboxylate (**1**):

At 60 °C, a solution of (3,4-dichlorophenyl)hydrazine hydrochloride (1.90 g, 8.92 mmol) in methanol (20 mL) was added slowly to a mixture of 1,2-cyclohexanedione (2.0 g, 17.84 mmol) in acetic acid (44 mL) and concentrated hydrochloric acid (16 mL). The reaction mixture was stirred for 3 h at 60 °C. The reaction mixture was then diluted with ethyl acetate and washed with water followed by brine. The organic layer was then dried over sodium sulfate and concentrated under reduced pressure. The residue was purified on an ISCO chromatograph (0–20% ethyl acetate/hexane) to give a mixture of two regio-isomers, 5,6-dichloro-2,3,4,9-tetrahydro-1*H*-carbazol-1-one (**1**) and 6,7-dichloro-2,3,4,9-tetrahydro-1*H*-carbazol-1-one (**2**) as a white solid (797 mg, 18%).

To a solution of a mixture of two regioisomers (797 mg, 3.14 mmol) in tetrahydrofuran (20 mL), boc anhydride (1.37 g, 6.28 mmol) and DAMP (384 mg, 3.14 mmol) were added. The reaction mixture was stirred for 3 h. The reaction mixture was then diluted with ethyl acetate and washed with saturated ammonium chloride followed by brine. The organic layer was then dried over sodium sulfate and concentrated under reduced pressure. The residue was purified on an ISCO chromatograph (0–10% ethyl acetate/hexane) to give *tert*-butyl 5,6-dichloro-1-oxo-1,2,3,4-tetrahydro-9*H*-carbazole-9-carboxylate (**1**) as a white solid (467 mg, 42%) (^1^H NMR (300 MHz) (CDCl_3_) δ 7.90 (d, *J* = 9 Hz, 1H), 7.47 (d, *J* = 9 Hz, 1H), 3.33 (t, *J* = 6 Hz, 2H), 2.64 (t, *J* = 7 Hz, 2H), 2.29–2.00 (m, 2H), 1.56 (s, 9H)) as well as *tert*-butyl 6,7-dichloro-1-oxo-1,2,3,4-tetrahydro-9*H*-carbazole-9-carboxylate (**2**) as a white solid (503 mg, 45%) (^1^H NMR (300 MHz) (CDCl_3_) δ 8.23 (s, 1H), 7.68 (s, 1H), 2.91 (t, *J* = 6 Hz, 2H), 2.67 (t, *J* = 7 Hz, 2H), 2.29–2.21 (m, 2H), 1.62 (s, 9H)).

### 2.3. MIC and MBC Assays

MIC assays were performed in duplicate according to Clinical and Laboratory Standards Institute (CLSI) guidelines for broth microdilution assay [25]. Briefly, log-phase bacteria were added at a concentration of 5 × 10^5^ cells/mL to 96-well microtiter plates containing twofold serial dilutions of A22, CBR-4830, or TXH11106 in CAMH broth. Cultures were grown with shaking at 37 °C for 18 h, and bacterial growth was measured by recording optical density at 600 nm (OD_600_) using a VersaMax plate reader (Molecular Devices, San Jose, CA, USA). MICs values were defined as the lowest compound concentration where growth was inhibited by ≥90%.

MBC values for each compound were determined by plating (in duplicate) 50 µL of the wells corresponding to 1×, 2×, and 4× MIC on TSA plates. Plates were incubated at 37 °C for 24 h, and counts of CFU/mL were determined. MBC values were defined as the lowest compound concentration that yielded ≥3 logs of kill relative to the initial inoculum (5 × 10^5^ cells/mL).

### 2.4. Time-Dependent Kill Assay

Overnight bacterial cultures were diluted to a final concentration of 1 × 10^6^ cells/mL in 5 mL of CAMH broth. Cultures were serially diluted in sterile PBS and plated on duplicate TSA plates, which were then incubated at 37 °C for 24 h and counted to determine CFU/mL values at time zero. TXH11106 was then added to cultures of each bacterial strain at final a concentration of 0.5×, 1×, or 4× MIC, with the assay for *P. aeruginosa* also including a culture with TXH11106 added at 8× MIC. DMSO was added to a culture of each bacterial strain as a vehicle control. All cultures were grown with shaking at 37 °C, and aliquots were serially diluted and plated on duplicate TSA plates after 3, 6, 9, and 24 h of incubation. The TSA plates were then incubated for 24 h at 37 °C, whereupon CFU/mL counts were determined.

### 2.5. Assay for Frequency of Resistance (FOR)

FOR assays were performed using a large inoculum approach described previously [26]. Briefly, overnight cultures were concentrated to a final count of ≈10^10^ cells/mL, serially diluted in sterile PBS, plated on duplicate TSA plates, incubated for 24 h at 37 °C, and then counted to determine the total number of plated cells. For the purposes of determining FOR, concentrated cultures were directly plated on duplicate TSA plates containing TXH11106 at 4× MIC (for *E. coli*, *K. pneumoniae*, and *A. baumannii*) or 8× MIC (for *P. aeruginosa*). The plates were then incubated at 37 °C for 48 h and counted. In the absence of any observed colonies on the TXH11106-containing plates, the FOR was defined as ≤(total number of plated cells)^−1^.

### 2.6. Differential Interference Contrast (DIC) Microscopy

Log-phase bacteria were diluted to 0.1 OD_600_ in 5 mL of CAMH broth, and TXH11106 was then added at a concentration of 1× MIC. An equivalent volume of DMSO was added to a separate culture as a vehicle control. The cultures were incubated with shaking for 3 h at 37 °C. A total of 1 to 5 mL of culture was pelleted at 15,000× *g* and washed twice with 1 mL of PBS. The pellets were then resuspended in 200 µL of PBS, and 8 µL was overlaid on pads of 1% agarose (in PBS) affixed to microscope slides. The bacteria were visualized by DIC microscopy using an Olympus BX50 microscope equipped with a 100× Olympus UPLSAPO oil immersion objective with a 1.40 aperture. The resulting images were captured using a QImaging Retiga R3 camera and processed using the Ocular version 2.0 software package (Teledyne Photometrics, Tuscon, AZ, USA). Image analysis and quantification was performed using the Fiji ImageJ software package.

The DIC microscopy results were quantified by determining the percentage of cells exhibiting a particular phenotype or morphology. Each percentage reflected an average of 5 different fields of view, with the average number of cells in each field of view ranging from 70 to 255 in the vehicle-treated groups and 110 to 258 for TXH11106-treated groups. The statistical significance of differences in cell morphology were analyzed using a one-way ANOVA test, with **** reflecting a *p*-value < 0.0001.

### 2.7. Cloning, Expression, and Purification of Wild-Type (WT) and E143G Mutant E. coli MreB

The *mreB* gene encoded by *E. coli* ATCC 25922 was PCR amplified from genomic DNA. Primers were designed to introduce an 8× His tag to the *N*-terminus of *E. coli* MreB (EcMreB). The PCR product was cloned into a pET-22b(+) plasmid (Novagen-Sigma Aldrich, St. Louis, MO, USA) using the NEBuilder Hifi DNA assembly cloning kit (New England Biolabs, Ipswich, MA, USA) and transformed into NEB 5a electrocompetent *E. coli* cells (New England Biolabs). Transformed cells were plated on LB agar containing 100 μg/mL ampicillin and incubated at 37 °C overnight. Individual colonies were isolated and grown overnight in 5 mL of LB broth containing 100 μg/mL ampicillin. Plasmids were then isolated using the Monarch plasmid miniprep kit (New England Biolabs), and their sequences were verified by Sanger sequencing. The plasmids were then transformed into calcium competent *E. coli* BL21(DE3) cells.

Transformed cells were grown on LB agar plates containing 100 μg/mL ampicillin, and individual colonies were used to inoculate 50 mL of ampicillin-containing LB broth. The culture was grown at 37 °C overnight, with 40 mL being subsequently added to 4 L of ampicillin-containing LB broth. The resulting culture was incubated until it reached an OD_600_ of 0.6, induced with isopropyl β-d-1-thiogalactopyranoside (IPTG) at a final concentration of 1 mM, and incubated at 15 °C for 18 h. The cells were then pelleted at 5000× *g* and 4 °C for 15 min and subsequently resuspended in 10 mL of ice-cold Buffer A (50 mM Tris-HCl pH 8.0, 50 mM KCl, 10 mM imidazole, and 10% glycerol). The cells were lysed by ultrasonication on ice for 20 min at 60 W with an on/off cycle of 10 s using a Qsonica Q500 sonicator equipped with a 0.5-inch probe. Cell lysates were centrifuged at 10,000× *g* and 4 °C for 30 min, added to 7 mL of TALON metal affinity resin (Clonetech Laboratories-Takara Bio USA, San Jose, CA, USA), and then incubated with gentle shaking at 4 °C for 1 h. The resin was washed with 50 mL of Buffer A and then added to a gravity flow column. A total of 15 mL of elution buffer (50 mM Tris-HCl pH 8.0, 50 mM KCl, 250 mM imidazole, and 10% glycerol) was added, and 500 μL fractions were collected at 4 °C. MreB-containing fractions were determined by SDS-PAGE and then dialyzed versus 4 L of dialysis buffer (50 mM Tris-HCl pH 8.0, 50 mM KCl, and 10% glycerol) with gentle stirring at 4°C overnight. Dialysates were concentrated using an Amicon Ultra-4 Ultracel 10K centrifugal filter (MilliporeSigma, Burlington, MA, USA), and protein concentration was determined with a Pierce BCA protein assay kit (Thermo Fisher Scientific, Waltham, MA, USA).

To generate E143G mutant EcMreB, primers were designed to introduce a GAA to GGT mutation at the relevant amino acid residue in the pET-22b(+)-*mreB* plasmid described above. Mutagenesis was performed using the site-directed mutagenesis kit from New England Biolabs, and the mutation was verified by sequence analysis. The pET-22b(+)-*mreB*(E143G) plasmid was then transformed into calcium-competent BL21(DE3) cells, and the E143G mutant EcMreB protein was expressed and purified as described above.

### 2.8. ATPase Assays

The ATPase activity of purified WT and E143G mutant EcMreB was characterized using an ATPase assay kit (Sigma-Aldrich). Briefly, 40 μL reactions in 96-well microtiter plates were initiated by adding 30 μL of 1.33 mM ATP to 10 μL of sample containing 8 µM WT EcMreB and differing concentrations of test compound (TXH11106, CBR-4830, or A22). The final concentrations in each reaction well were 0 to 1000 μM compound, 2 μM EcMreB, and 1 mM ATP. Final reaction buffer conditions were 20 mM Tris-HCl pH 7.5, 40 mM NaCl, 4 mM magnesium acetate, and 0.5 mM EDTA. Negative controls included DMSO vehicle instead of compound and MreB dialysis buffer instead of protein (no protein background). A 0 to 50 μM phosphate standard curve was prepared to calculate the phosphate production in each well. All samples, controls, and standards were prepared in duplicate, and the reactions were run at room temperature for 30 min. After 30 min, the reactions were stopped by the addition of 200 μL of malachite green reagent, and the colorimetric product was measured at 620 nm using a SpectraMax M2 plate reader (Molecular Devices). Final OD_620_ readings were corrected for the no protein background, and the standard curve was used to determine the total amount of phosphate produced by each reaction. Reaction velocities were determined from the phosphate produced over the course of 30 min. Compound concentrations that inhibit MreB ATPase activity by 50% (IC_50_ values) were determined by fitting plots of reaction velocity (*v*) versus compound concentration © with the following relationship:

(1) $$v=\frac{v_{max}}{1+{\left(\frac{C}{{\mathrm{IC}}_{50}}\right)}^{m}}$$

In this equation, *v_max_* is the maximal (uninhibited) reaction velocity and *m* is the Hill slope. The same assay was used to determine the impact of TXH11106 on the ATPase activity of E143G mutant EcMreB.

To analyze the impact of A22, CBR-4830, and TXH11106 on the catalytic activity of EcMreB, the reactions were set up and run as described above, except that compound concentrations were maintained at 30 μM and the final ATP concentrations ranged from 0.1 to 1 mM. Separate backgrounds were run for each concentration of ATP. All sample and control reactions were run in triplicate. At each ATP concentration, velocities were determined as described above, and catalytic parameters were calculated from the resulting velocities. The impact of 30 μM TXH11106 on the catalytic activity of E143G mutant EcMreB was determined as described above.

## 3. Results

### 3.1. Bactericidal Activity of TXH11106 against the Gram-Negative Pathogens E. coli, K. pneumoniae, A. baumannii, and P. aeruginosa

We compared the minimal inhibitory concentration (MIC) and minimal bactericidal concentration (MBC) of TXH11106 relative to CBR-4830 and A22 against the Gram-negative pathogens *E. coli* (ATCC 25922), *K. pneumoniae* (ATCC 13883), *A. baumannii* (ATCC 19606), and *P. aeruginosa* (ATCC 27853). The MIC values of TXH11106 against these pathogens ranged from 4 µg/mL (versus *E. coli*) to 8 µg/mL (versus *K. pneumoniae*, *A. baumannii*, and *P. aeruginosa*), with these values being two- to fourfold lower than the corresponding values for CBR-4830 and 4- to 64-fold lower than the corresponding values for A22 (Table 1). TXH11106 was also associated with MBC values of 4 µg/mL versus *E. coli*, 8 µg/mL versus *K. pneumoniae*, 16 µg/mL versus *A. baumannii*, and 32 µg/mL versus *P. aeruginosa* (Table 1). The corresponding MBC values of CBR-4830 were twofold higher versus *E. coli* and four- to eightfold higher versus the other three strains. A22 was associated with the highest MBC values of all, ranging from 32 µg/mL versus *E. coli* to >256 µg/mL versus *P. aeruginosa*.

**Figure 2:**
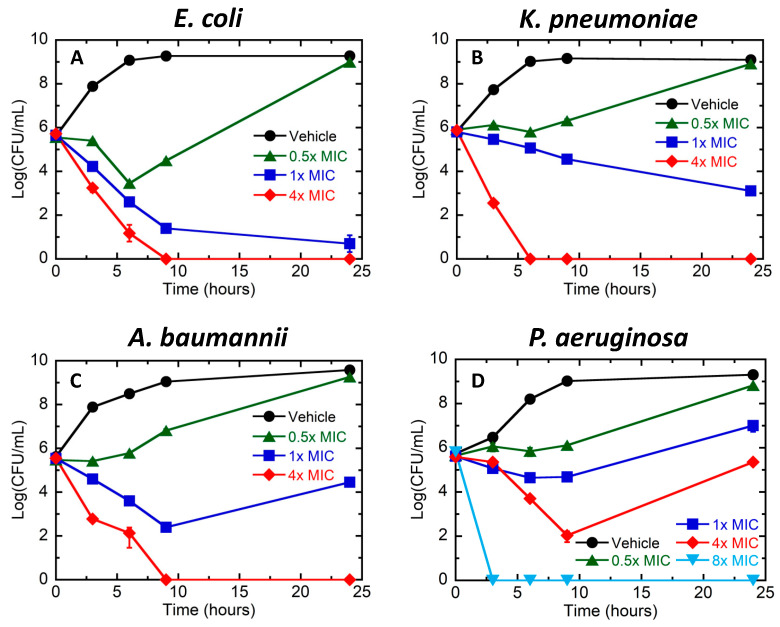
Time–kill curves for *E. coli* 25922 (**A**), *K. pneumoniae* 13883 (**B**), *A. baumannii* 19606 (**C**), and *P. aeruginosa* 27853 (**D**) treated with DMSO vehicle or TXH11106 at the indicated concentrations. Each experimental data point represents the average of two replicates, with the error bars reflecting the standard deviation from the mean.

We further characterized the bactericidal activity of TXH11106 by measuring the time-dependent killing of *E. coli*, *K. pneumoniae*, *A. baumannii*, and *P. aeruginosa* induced by differing concentrations of TXH11106. At 1× MIC, 24 h of treatment induced approximately 5 logs of kill in *E. coli* and 3 logs of kill in *K. pneumoniae* (Figure 2A,B). Treatment of both strains at 4× MIC resulted in total kill within 6–9 h. In *A. baumannii*, treatment at 1× MIC induced 4 logs of kill in 9 h but was followed by 2 logs of growth between 9 and 24 h (Figure 2C). However, treatment at 4× MIC resulted in total kill within 9 h, a behavior similar to that observed in *E. coli* and *K. pneumoniae*. In *P. aeruginosa*, treatment at 1× MIC inhibited growth by approximately 2 logs but did not kill (Figure 2D). At 4× MIC, the time–kill pattern in *P. aeruginosa* was comparable to that observed in *A. baumannii* treatment at 1× MIC. Treatment of *P. aeruginosa* at 8× MIC resulted in total kill within 3 h.

### 3.2. Frequency of Resistance (FOR) in E. coli, K. pneumoniae, A. baumannii, and P. aeruginosa Following Exposure to TXH11106

We next probed for the emergence of mutational resistance in large inocula (≈10^9^–10^10^ CFU) of *E. coli*, *K. pneumoniae*, *A. baumannii*, and *P. aeruginosa* following 48 h of exposure to TXH11106 at concentrations yielding total time-dependent kill (4× or 8× MIC). In *E. coli*, *K. pneumoniae*, and *A. baumannii*, no detectable colonies were observed with TXH11106 exposure at 4× MIC, resulting in FOR values ranging from <6.2 × 10^−10^ to <1.3 × 10^−9^ (Table 2). A similar behavior was observed in *P. aeruginosa* with exposure to TXH1106 at 8× MIC, where an FOR of <1.0 × 10^−9^ was determined (Table 2).

### 3.3. Impact of TXH11106 Treatment on the Morphology of E. coli, K. pneumoniae, A. baumannii, and P. aeruginosa

**Figure 3:**
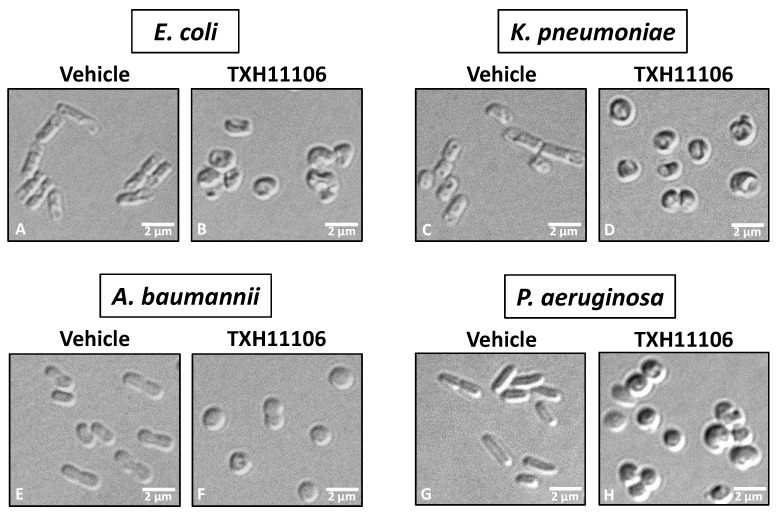
Differential interference contrast (DIC) micrographs of *E. coli* 25922 (**A**,**B**), *K. pneumoniae* 13883 (**C**,**D**), *A. baumannii* 19606 (**E**,**F**), and *P. aeruginosa* 27853 (**G**,**H**) treated for 3 h with DMSO vehicle (**A**,**C**,**E**,**G**) or TXH11106 at 1× MIC (**B**,**D**,**F**,**H**). The scale bars reflect 2 µm in length.

**Figure 4:**
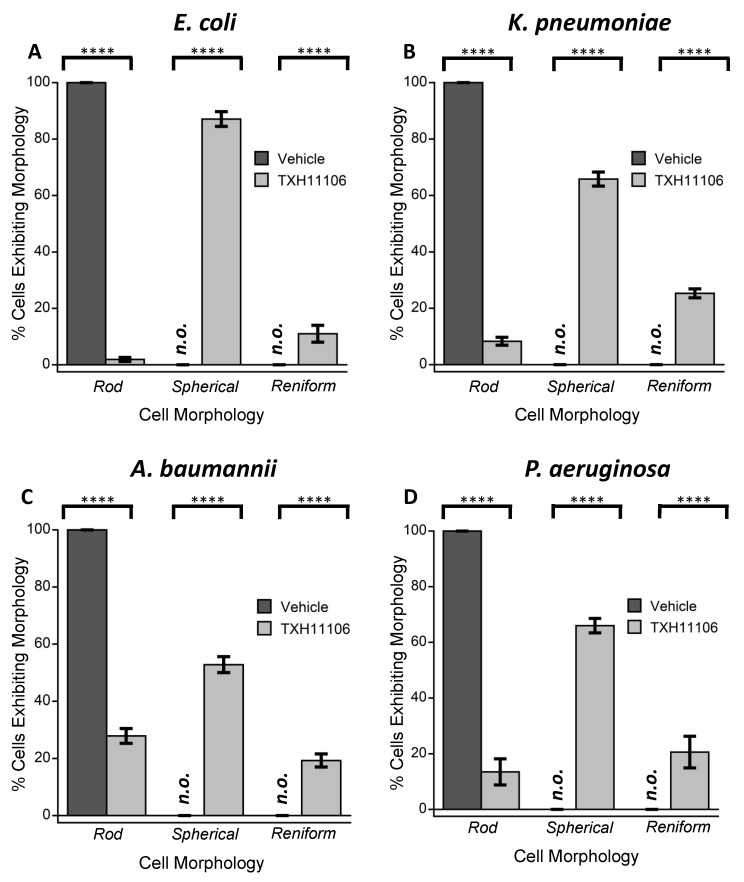
Quantification of the cell morphology results from the DIC microscopy experiments depicted in Figure 3. The bar graphs show the prevalence (in %) of the various cell morphologies (rod, spherical, and reniform) observed in the vehicle-treated groups (*n* ranging from 349 to 1277) and TXH11106-treated groups (*n* ranging from 550 to 1291) for *E. coli* 25922 (**A**), *K. pneumoniae* 13883 (**B**), *A. baumannii* 19606 (**C**), and *P. aeruginosa* 27853 (**D**). Each percentage reflects an average of 5 different fields of view, with the average number of cells in each field of view ranging from 70 to 255 in the vehicle-treated groups and 110 to 258 for TXH11106-treated groups. The indicated error bars reflect the standard deviation from the mean. The statistical significance of differences in cell morphology were analyzed using a one-way ANOVA test. **** reflects a *p*-value < 0.0001. n.o. = none observed.

As an initial characterization of the mechanism underlying the bactericidal activity of TXH11106, we used differential interference contrast (DIC) microscopy to examine the impact of TXH11106 treatment (at 1× MIC) for 3 h on the morphology of *E. coli*, *K. pneumoniae*, *A. baumannii*, and *P. aeruginosa*. Upon treatment with DMSO vehicle, 100% of the cells examined (*n* ranging from 349 to 1277) exhibited their expected rod shapes (Figure 3A,C,E,G and Figure 4), with many septa being evident. In marked contrast, treatment with TXH11106 resulted in the majority (52.8–87.1%) of examined cells (*n* ranging from 550 to 1291) being spherical in shape and much fewer (1.9–27.9%) being rod-shaped (Figure 3B,D,F,H and Figure 4). The remainder of the TXH11106-treated cells (11.0–25.3%) were reniform in shape. These morphological changes induced by TXH11106 treatment are consistent with an MreB-directed mode of antibacterial action [21].

### 3.4. TXH11106-Induced Inhibition of the ATPase Activity of E. coli MreB (EcMreB)

**Figure 5:**
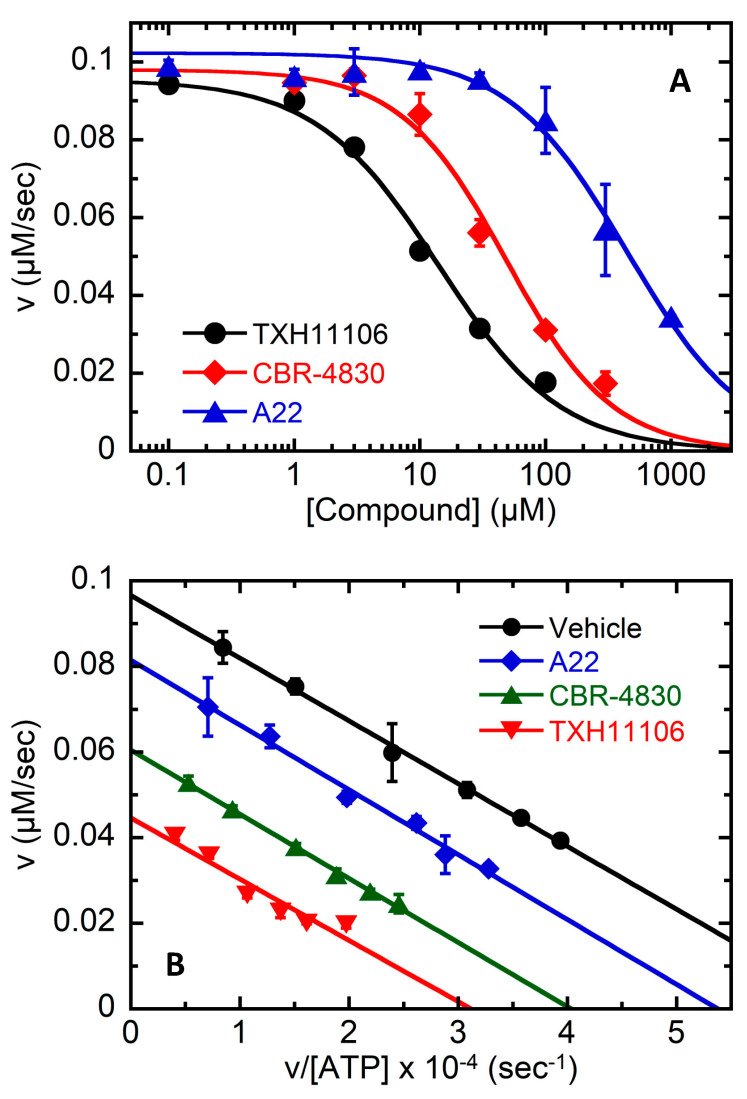
(**A**) Impact of increasing concentrations of TXH11106, CBR-4830, or A22 on the velocity of the ATPase reaction of wild-type (WT) *E. coli* MreB (EcMreB). Each experimental data point represents an average of two replicates, with the indicated error bars reflecting the standard deviation from the mean. The solid curves represent nonlinear least squares fits of the experimental data points with Equation (1). (**B**) Eadie–Hofstee plots of the ATP-dependent catalytic activity of WT EcMreB in the presence of DMSO vehicle or 30 µM inhibitor (TXH11106, CBR-4830, or A22). Each experimental data point represents an average of 3 replicates, with the indicated error bars reflecting the standard deviation from the mean. The solid lines represent linear fits of the experimental data points.

To further validate MreB as the antibacterial target of TXH11106, we examined its comparative impact on the ATPase activity of purified wild-type (WT) EcMreB relative to A22 and CBR-4830. Like A22 and CBR-4830, TXH11106 inhibited the ATPase activity of EcMreB, as demonstrated by the concentration-dependent velocity profiles shown in Figure 5A. Analysis of these plots yielded IC_50_ values of 14 ± 2, 49 ± 8, and 447 ± 87 µM for TXH11106, CBR-4830, and A22, respectively (Table 3). Thus, TXH11106 inhibited EcMreB with a 3.5-fold lower IC_50_ than CBR-4830 and a 32-fold lower IC_50_ than A22.

To determine the mechanism of inhibition, we also compared how TXH11106, CBR-4830, and A22 impact the kinetics of WT EcMreB ATPase activity by examining catalysis as a function of ATP concentration in the absence and presence of 30 µM inhibitor. The results of these characterizations are presented in the form of the Eadie–Hofstee plots shown in Figure 5B. Analysis of these plots produced the catalytic parameters listed in Table 4. Inspection of these data reveals that treatment with the inhibitors did not significantly impact K_m_ (K_m_ = 143 ± 21, 150 ± 4, 151 ± 8 µM for TXH11106, CBR-4830, and A22, respectively) compared to treatment with DMSO vehicle (K_m_ = 147 ± 4 µM). By contrast, inhibitor treatment decreased both V_max_ (from (9.66 ± 0.10) × 10^−2^ µM/s for DMSO vehicle to (4.46 ± 0.28) × 10^−2^, (6.05 ± 0.07) × 10^−2^, and (8.15 ± 0.19) × 10^−2^ µM/s for TXH11106, CBR-4830, and A22, respectively) and k_cat_ (from (4.83 ± 0.05) × 10^−2^ s^−1^ for DMSO vehicle to (2.23 ± 0.14) × 10^−2^, (3.03 ± 0.04) × 10^−2^, and (4.08 ± 0.10) × 10^−2^ s^−1^ for TXH11106, CBR-4830, and A22, respectively). These collective effects are consistent with a non-competitive mechanism of inhibition. In addition, the impact of inhibitor treatment on k_cat_ and K_m_ yielded a corresponding decrease in k_cat_/K_m_ from (3.29 ± 0.11) × 10^−4^ µM^−1^s^−1^ for vehicle to (1.56 ± 0.33) × 10^−4^, (2.02 ± 0.08) × 10^−4^, and (2.70 ± 0.21) × 10^−4^ µM^−1^s^−1^ for TXH11106, CBR-4830, and A22, respectively. Thus, at an equivalent concentration of 30 µM, TXH11106 reduced the k_cat_/K_m_ of WT EcMreB by 2.1-fold, compared to only 1.6-fold for CBR-4830 and 1.2-fold for A22.

### 3.5. Impact of the E143G Mutation on the Catalytic Activity of EcMreB as Well as Inhibition by TXH11106

**Figure 6:**
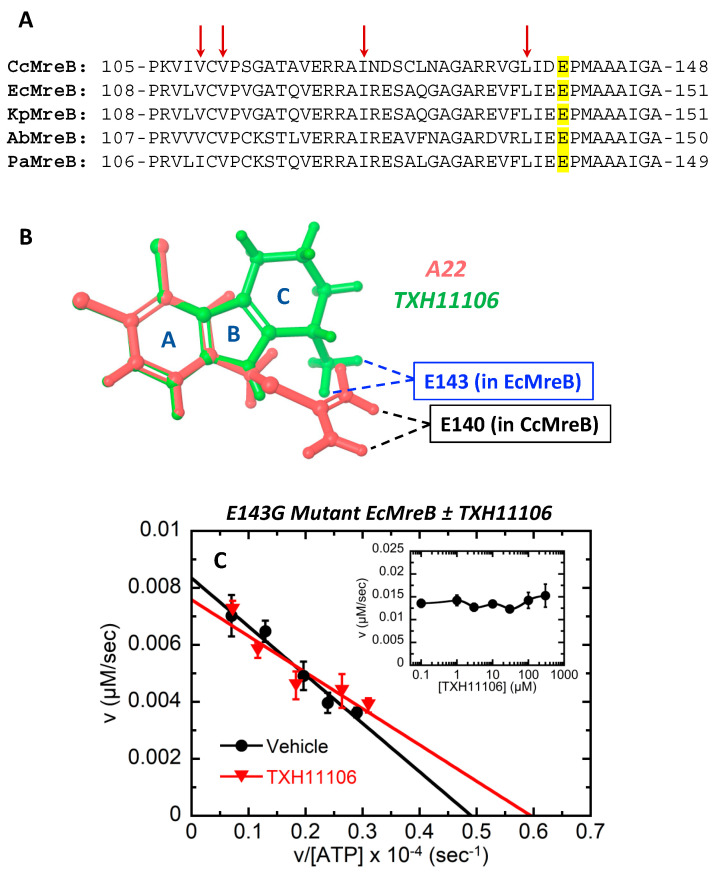
(**A**) Alignment of a portion of the amino acid sequence from *C. crescentus* MreB (CcMreB), *E. coli* MreB (EcMreB), *K. pneumoniae* MreB (KpMreB), *A. baumannii* MreB (AbMreB), and *P. aeruginosa* MreB (PaMreB). As revealed by the previously reported crystal structure of the CcMreB-A22 complex (PDB: 4CZG) [23], this portion of the MreB amino acid sequence includes residues that form key contacts with the inhibitor. The E140 residue in CcMreB (highlighted in yellow) that forms electrostatic and hydrogen bonding contacts with the amidine functionality of A22 is conserved in the EcMreB, KpMreB, AbMreB, and PaMreB proteins. The hydrophobic residues of CcMreB (indicated by the arrows) that form Van der Waals contacts with ring A of A22 are also conserved in the other four MreB proteins. (**B**) Overlay of the gas-phase models of A22 (red) and TXH11106 (green). The contacts observed between the E140 residue of CcMreB and the amidine substituent of A22 are depicted by the black dashed lines, with the proposed corresponding contacts between the E143 residue of EcMreB and the amino functionality on ring C of TXH11106 being depicted by the blue dashed lines. The gas-phase models were generated using the Jaguar application of the Schrodinger Maestro version 12.9.123 software package. Compound geometries were optimized in the functional B3LYP-D3 gas phase. (**C**) Eadie–Hofstee plots of the ATP-dependent catalytic activity of E143G mutant EcMreB in the presence of DMSO vehicle or 30 µM TXH11106. Each experimental data point represents an average of three replicates, with the indicated error bars reflecting the standard deviation from the mean. The solid lines represent linear fits of the experimental data points. The inset depicts the impact of increasing concentrations of TXH11106 on the velocity of the ATPase reaction of E143G mutant EcMreB. Each experimental data point represents an average of two replicates, with the indicated error bars reflecting the standard deviation from the mean.

The Glu residue E143 in EcMreB is highly conserved among the MreB proteins of most rod-shaped Gram-negative pathogens, including *K. pneumoniae* (E143 in KpMreB), *A. baumannii* (E142 in AbMreB), and *P. aeruginosa* (E141 in PaMreB). These conserved Glu residues are highlighted in yellow in Figure 6A. In the crystal structure of A22 in complex with CcMreB (PDB: 4CZG) reported by Löwe and coworkers [23], strong electrostatic and hydrogen bonding contacts were observed between the corresponding Glu residue in CcMreB (E140) and the amidine functionality of A22 (schematically depicted in Figure 6B). An overlay of the gas-phase structures of TXH11106 and A22 suggests that the amino functionality on the cyclohexyl ring (ring C) of TXH11106 would form similar contacts with the E143 residue of EcMreB (Figure 6B). To test this hypothesis, we cloned and expressed an E143G mutant form of EcMreB and evaluated the impact of this mutation on both the catalytic activity of the protein and the inhibitory activity of TXH11106.

Eadie–Hofstee plots of the ATP-dependent catalytic activity of E143G mutant EcMreB upon treatment with DMSO vehicle or 30 µM TXH11106 are shown in Figure 6C. Analysis of the vehicle treatment plot revealed that E143G mutant EcMreB was associated with much reduced V_max_ ((0.84 ± 0.04) × 10^−2^ µM/s), k_cat_ ((0.42 ± 0.02) × 10^−2^ s^−1^), and k_cat_/K_m_ ((0.25 ± 0.04) × 10^−4^ µM^−1^s^−1^) values compared to vehicle-treated WT EcMreB (Table 4). Thus, the E143G mutation impaired the catalytic efficiency of EcMreB. Significantly, the Eadie–Hofstee plots for both vehicle and TXH11106 treatment of E143G mutant EcMreB were very similar (Figure 6C), with 30 µM TXH11106 having little or no effect on the catalytic activity of the mutant protein (Table 4). Consistent with this observation, TXH11106 concentrations up to 300 µM did not significantly impact the catalytic velocity of E143G mutant EcMreB (Figure 6C inset), resulting in an IC_50_ > 300 µM (Table 3). Thus, in addition to reducing catalytic efficiency, the E143G mutation in EcMreB also abolished the inhibitory activity of TXH11106.

## 4. Discussion

We describe the antibacterial activity of the third-generation MreB inhibitor TXH11106 against the Gram-negative pathogens *E. coli*, *K. pneumoniae*, *A. baumannii*, and *P. aeruginosa*. Our MIC and MBC results revealed that the potency of TXH11106 is greatest versus *E. coli* (MIC = MBC = 4 µg/mL), followed by *K. pneumoniae* (MIC = MBC = 8 µg/mL), *A. baumannii* (MIC = 8 µg/mL, MBC = 16 µg/mL), and *P. aeruginosa* (MIC = 8 µg/mL, MBC = 32 µg/mL) (Table 1). The activity of TXH11106 against all four pathogens is bactericidal in nature, as reflected by MBC/MIC ratios ≤ 4. Time-dependent kill curves confirmed the bactericidal effects of TXH11106, with a concentration of 4× MIC resulting in total kill of *E. coli*, *K. pneumoniae*, and *A. baumannii* cells within 6–9 h (Figure 2A–C). A TXH11106 concentration of 8× MIC was required for total kill of *P. aeruginosa* cells, which occurred within 3 h (Figure 2D). While TXH11106 at 4× MIC killed >3 logs of *P. aeruginosa* cells within 9 h, grow back was observed between 9 and 24 h. The *mreb* sequences isolated from colonies that grew at 24 h were all wild type in nature (not shown), suggesting that the observed grow back did not reflect mutational resistance. Instead, the grow back may reflect a subpopulation of persister *P. aeruginosa* cells that became tolerant of TXH11106 at 4× MIC, perhaps by transitioning to an L-form. However, TXH11106 at 8× MIC was sufficient to prevent the emergence of persister subpopulations.

We compared the activity TXH11106 against *E. coli*, *K. pneumoniae*, *A. baumannii*, and *P. aeruginosa* relative to the corresponding activities exhibited by the first- and second-generation MreB inhibitors A22 and CBR-4830, respectively. Significantly, our MIC and MBC characterizations demonstrated that TXH11106 is two- to fourfold more potent than CBR-4830 and 4- to 64-fold more potent than A22 (Table 1). Given the enhanced activity of TXH11106, we sought to determine the potential mutational resistance to emerge against the compound in the presence of bacterial loads (≈10^9^–10^10^ CFUs) in the range that can be present in clinically relevant infections [27]. No evidence of mutational resistance was observed upon 48 h of exposure to TXH11106 at 4× MIC (in *E. coli*, *K. pneumoniae*, and *A. baumannii*) or 8× MIC (in *P. aeruginosa*).

As a first step toward validating MreB as the antibacterial target of TXH11106, we used DIC microscopy to explore the impact of TXH11106 treatment at 1× MIC for 3 h on the morphology of *E. coli*, *K. pneumoniae*, *A. baumannii*, and *P. aeruginosa* cells. In all four strains, treatment with TXH11106 induced a profound morphological change from the normal rod shape to a predominantly spherical shape (Figure 3 and Figure 4). These TXH11106-induced morphological changes are consistent with MreB inhibition, a similar behavior having been previously observed in *E. coli* and *P. aeruginosa* upon treatment with A22 and CBR-4830 [21,24].

We next sought to determine if the relative antibacterial potencies of TXH11106, CBR-4830, and A22 correlate with the relative abilities of the compounds to inhibit MreB ATPase activity. To this end, we cloned and expressed WT EcMreB and found that the relative IC_50_ values for TXH11106, CBR-4830, and A22 (14 ± 2, 49 ± 8, and 447 ± 87 µM, respectively) (Figure 5A and Table 3) correlate well with the relative antibacterial activities of the compounds (TXH11106 > CBR-4830 > A22) (Table 1). This gratifying concordance further validates MreB as the antibacterial target of TXH11106.

A22 was one of the first MreB inhibitors identified with activity against rod-shaped Gram-negative bacteria [21]. It consists of a halogen-substituted ring constituent (3,4-dichloro-substituted ring A) coupled to a basic thiourea constituent (Figure 1). Like A22, the second-generation agent CBR-4830 also has a halogen-substituted ring constituent (6-bromo-substituted ring A) conjugated via a second ring (ring B) to a basic ring functionality (1-amino-substituted ring C) (Figure 1). TXH11106 incorporates the dichloro substitution pattern on ring A of A22 into the three-ringed structure of CBR-4830. The crystal structure of CcMreB in complex with both A22 and ADP determined by Löwe and coworkers [23] revealed that the compound targets a site adjacent to the nucleotide binding pocket. This interaction was stabilized in part by electrostatic and hydrogen bonding contacts between the amidine portion of the A22 thiourea functionality and the Glu residue at position 140 (E140). This Glu residue is highly conserved among Gram-negative bacteria, including in *E. coli*, *K. pneumoniae*, *A. baumannii*, and *P. aeruginosa* (shaded in yellow in Figure 6A). An overlay of the gas-phase models of A22 and TXH11106 (Figure 6B) reveals that the amidine functionality of A22 and the amine substituent on ring C of TXH11106 adopted similar spatial orientations, with the same likely to be true of the amine group on ring C of CBR-4830. The enhanced inhibition of EcMreB by CBR-4830 and TXH11106 relative to A22 may reflect contributions from more optimized interactions with the conserved Glu residue in the binding site.

The crystal structure of the A22–CcMreB complex also revealed the presence of Van der Waals contacts between the dichloro substituents on ring A of the compound and hydrophobic residues lining the binding site (including V109, V111, I123, and L137) that are also highly conserved among Gram-negative bacteria (as reflected by the arrows in Figure 6A). Such contacts are thus likely to be present in the interaction of EcMreB and TXH11106, which like A22, also has dichloro substituents on ring A. These dichloro functionalities may afford enhanced Van der Waals contacts with EcMreB relative to the bromo substituent on ring A of CBR-4830, thereby conferring TXH11106 with an enhanced inhibitory activity. In this connection, one of the key motivating factors for incorporation of the dichloro substituents on ring A of TXH11106 was to improve the Van der Waals interactions between this ring and the target MreB protein.

To determine the mechanism with which TXH11106 inhibits the ATPase activity of WT EcMreB, we evaluated the impact of the compound on the kinetics of ATP-dependent catalysis by EcMreB. The resulting Eadie–Hofstee plot for TXH11106 was parallel relative to that for DMSO vehicle (Figure 5B), indicative of a non-competitive mechanism of inhibition [28]. We observed a similar behavior in comparator characterizations of CBR-4830 and A22 (Figure 5B). These observations are consistent with the crystallographic results of Löwe and coworkers who found that both A22 and ADP were able to bind CcMreB in adjacent binding sites [23]. Catalytic parameters derived from analysis of the Eadie–Hofstee plots for TXH11106, CBR-4830, and A22 were also consistent with a non-competitive mechanism of inhibition, with K_m_ being essentially unchanged and both V_max_ and k_cat_ being decreased relative to vehicle (Table 4) [28]. k_cat_/K_m_ ratios calculated from these parameters were also decreased for all three compounds relative to vehicle (Table 4). In addition, the magnitudes of the k_cat_ and k_cat_/K_m_ values for the three compounds followed the hierarchy TXH11106 < CBR-4830 < A22. Taken together, these results indicate that all three compounds non-competitively inhibit EcMreB by reducing its catalytic efficiency, with TXH11106 inhibiting catalysis to the greatest extent followed by CBR-4830 and A22.

On the basis of their crystal structure of A22 and ADP in complex with CcMreB, Löwe and coworkers hypothesized that the conserved Glu residue E140 (E143 in EcMreB) may play a key role in the catalysis of ATP hydrolysis. To test this hypothesis, we cloned and expressed an E143G mutant form of EcMreB and evaluated its ability to catalyze ATP hydrolysis compared to WT EcMreB. Significantly, E143G mutant EcMreB hydrolyzed ATP with a 13-fold reduced catalytic efficiency compared to WT EcMreB (Figure 6C and Table 4), confirming Löwe’s hypothesis. Löwe has further suggested that A22 may inhibit MreB by interacting with the conserved Glu residue and inhibiting its catalytic function [23]. Consistent with this hypothesis, TXH11106 exerts a negligible impact on the catalytic activity of E143G mutant EcMreB (Figure 6C, Table 4). This observation suggests that TXH11106 and, by extension, CBR-4830 and A22, may inhibit EcMreB ATPase activity by blocking the catalytic function of E143.

## 5. Conclusions

Here, we show that the third-generation MreB inhibitor TXH11106 is associated with enhanced bactericidal activity against the clinically significant Gram-negative pathogens *E. coli*, *K. pneumoniae*, *A. baumannii*, and *P. aeruginosa* relative to the first- and second-generation agents A22 and CBR-4830, respectively. Enzymatic studies with WT and E143G mutant EcMreB indicate that the enhanced antibacterial potency of TXH11106 is correlated with a correspondingly enhanced ability to inhibit the catalytic efficiency MreB, with this inhibition being non-competitive in nature. Morphological changes induced by TXH11106 in *E. coli*, *K. pneumoniae*, *A. baumannii*, and *P. aeruginosa* further validate MreB as the antibacterial target of the compound. Large inocula (≈10^9^–10^10^ CFUs) of these pathogens do not produce mutational resistance to TXH11106 with 48 h of exposure. Viewed as a whole, our results highlight TXH11106 as a lead MreB inhibitor of interest.

## Figures and Tables

**Table 1:** Activity of TXH11106 and comparator MreB inhibitors against the Gram-negative pathogens *E. coli*, *K. pneumoniae*, *A. baumannii*, and *P. aeruginosa*.

Compound	Antibacterial Parameters (MIC and MBC Listed in µg/mL)											
	*E. coli* 25922			*K. pneumoniae* 13883			*A. baumannii* 19606			*P. aeruginosa* 27853		
	MIC	MBC	$\frac{\mathrm{MBC}}{\mathrm{MIC}}$	MIC	MBC	$\frac{\mathrm{MBC}}{\mathrm{MIC}}$	MIC	MBC	$\frac{\mathrm{MBC}}{\mathrm{MIC}}$	MIC	MBC	$\frac{\mathrm{MBC}}{\mathrm{MIC}}$
TXH11106	4	4	1	8	8	1	8	16	2	8	32	4
CBR-4830	8	8	1	16	64	4	32	64	2	32	128	4
A22	256	256	1	128	256	2	32	128	4	32	>256	>8

MIC = minimum inhibitory concentration; MBC = minimum bactericidal concentration. An MBC/MIC ratio of ≤4 is reflective of a bactericidal mode of action, while an MBC/MIC ratio of >4 is reflective of a bacteriostatic mode of action.

**Table 2:** Frequency of resistance (FOR) in *E. coli*, *K. pneumoniae*, *A. baumannii*, and *P. aeruginosa* upon exposure to THX11106.

Strain	[TXH11106] (µg/mL)	FOR
*E. coli* 25922	16	<6.2 × 10^−10^
*K. pneumoniae* 13883	32	<8.2 × 10^−10^
*A. baumannii* 19606	32	<1.3 × 10^−9^
*P. aeruginosa* 27853	64	<1.0 × 10^−9^

The indicated concentrations of TXH11106 reflect 4× MIC for *E. coli*, *K. pneumoniae*, and *A. baumannii*, and 8× MIC for *P. aeruginosa*.

**Table 3:** IC_50_ values for inhibition of the ATPase activity of wild-type (WT) and E143G mutant EcMreB.

Compound	MreB	IC_50_ (μM)
TXH11106	WT	14 ± 2
CBR-4830	WT	49 ± 8
A22	WT	447 ± 87
TXH11106	E143G	>300

EcMreB = *E. coli* MreB; IC_50_ = the compound concentration that inhibits the ATPase activity of EcMreB by 50%. IC_50_ values for WT EcMreB were derived from fits of the velocity vs. [TXH11106] plots shown in Figure 5A with Equation (1), with the indicated uncertainties reflecting the standard deviation of the fitted curves from the experimental data points. The IC_50_ value for E143G mutant EcMreB was derived from the velocity (*v*) vs. [TXH11106] plot shown in the inset of Figure 6C.

**Table 4:** Enzyme kinetics for the ATPase reaction of wild-type (WT) and E143G mutant EcMreB in the absence and presence of 30 µM inhibitor.

Sample	MreB	K_m_ (μM)	V_max_ × 10^2^ (μM/s)	k_cat_ × 10^2^ (s^−1^)	k_cat_/K_m_ × 10^4^ (μM^−1^s^−1^)
Vehicle	WT	147 ± 4	9.66 ± 0.10	4.83 ± 0.05	3.29 ± 0.11
TXH11106	WT	143 ± 21	4.46 ± 0.28	2.23 ± 0.14	1.56 ± 0.33
CBR-4830	WT	150 ± 4	6.05 ± 0.07	3.03 ± 0.04	2.02 ± 0.08
A22	WT	151 ± 8	8.15 ± 0.19	4.08 ± 0.10	2.70 ± 0.21
Vehicle	E143G	170 ± 18	0.84 ± 0.04	0.42 ± 0.02	0.25 ± 0.04
TXH11106	E143G	127 ± 28	0.76 ± 0.06	0.38 ± 0.03	0.30 ± 0.09

K_m_ = Michaelis constant; V_max_ = maximum velocity; k_cat_ = turnover number; k_cat_/K_m_ = catalytic efficiency. Values of K_m_ and V_max_ for WT and E143G mutant EcMreB were derived from linear fits of the Eadie–Hofstee plots shown in Figure 5B and Figure 6C, respectively, with the indicated uncertainties reflecting the standard deviation of the fitted lines from the experimental data points. Values of k_cat_ were determined from V_max_/[EcMreB]_total_, with the indicated uncertainties reflecting the propagation of errors through that ratio. The indicated uncertainties for the values of k_cat_/K_m_ reflect the propagation of errors through that ratio.

## Data Availability

Not applicable.
